# Magnetic and Electric Forces

**Published:** 1886-11

**Authors:** 


### MAGNETIC AND ELECTRICAL FORCES.

The following matter is copied by permission in advance from a work called *Human Culture and Cure* by E. D. Babbitt, M. D., the first number of which is soon to be issued. This work aims to develop a science of Therapeutics and of human upbuilding based on the great underlying laws of the chemical, electrical, magnetic, luminous, physiological and psychological forces of nature.

MAGNETISM, as Ampere has shown, is merely *electricity thrown into curves.* A magnet has countless millions of electrical streams drawn around and around into whirlwinds of force by the tremendous suctional power of the vortexes in the atoms of steel through which they pass, and has also a series of streams that are not thus deflected into curves, the strongest of which currents enter at the negative or south pole and emerge at the positive or north pole, while other and weaker grades of electrical ethers pass through the magnet in just the opposite direction. The greatest magnetic tension is at or near the poles, there being no force in the middle of a magnet strong enough to control iron filings. The horseshoe magnet is more powerful than a bar magnet because the suction of both poles can be used. If the positive poles of two magnets are placed together their powerful streams of ether will strike against each other, and drive each other apart. This is the philosophy of REPULSION and explains the well-known expression *similaros repel.* If a positive and negative end are placed together the electrical currents of both will flow in the same direction and *intensify each other*, causing them to rush together with great force. This is the explanation of ATTRACTION and exemplifies the law expressed by the words *contrasts* attract. A hundred mysteries are explained by this etherio-atomic law of force which thus far has not been understood.

All substances contain some grade of magnetism, or at least of Diamagnetism, in which latter the ethers pass through objects transversely instead of longitudinally and work more feebly.

The ordinary magnetism which works with iron and steel is Ferro magnetism; another grade which is connected with animal life is called Animal Magnetism; a higher and finer grade of ethers connected with the mental, spiritual and intuitional nature is called Psycho-magnetism or Psychaura, which is literally *soul force*. The term Vital Magnetism may be used to include both animal and psycho-magnetism. In Woman the forces are more influx or negative; in man they are more efflux or positive. For this reason, according to the law, already explained, it is conducive to the harmony and activity of the vital forces for the sexes to be frequently in the same atmosphere with each other in school-rooms, churches, societies and social circles and is also for the greater purity of both. All monastical and ascetic exclusion of the sexes is founded on ignorance, and leads in the end to lower conditions, as well as to greater misery. Solar Magnetism animates and develops much of the vegetable and animal life of the world and like psycho-magnetism has a wonderful potency in the cure of disease.

Terrestrial Electricities. We can never enjoy perfect health until we learn to live in harmony with the forces of the earth. Baron Reichenbach in his numerous and remarkable experiments with reference to Odic force, ascertained that multitudes of sensitive persons were having their nervous systems wrecked and even the power of medicines to a great extent destroyed by sleeping with their heads to the south or the west. He found that by placing them with their heads to the north they became relieved at once. Some were so very sensitive that they almost went into spasms when placed only a few minutes to the south or west. Very many cases in proof of this law have come under my own observation. The explanation of this matter, which the Baron did not seem to understand, and which our scientists do not understand even yet, is as follows: The magnetic poles which are culminating points of cold and tremendous vortexes of the electricities that are ever rushing inward toward the region of the earth's interior to bring about an equilibrium, draw the electricities from all directions northward toward themselves in the northern hemisphere, and southward toward their culminating points in the southern hemisphere. These northward electricities which drive the magnetic needle northward are cold forces and we should lie with the head northward because it is the most charged with blood and heat and should catch the cooling principle. In the Southern hemisphere the head should lie southward for the same reason. When cold forces move in one direction the warm forces move in the other, and it is especially bad to have these warm currents moving upward through the nerves and spine toward the brain as they must do when the head is southward. A modification of these electricities is caused by the course of the sun which carries a warm tide of ethers westward in consequence of which a cool tide must flow eastward through the day and most of the night. For this reason a direction a little east of north in the northern hemisphere and a little east of south in the southern would doubless be best, as my own experience has actually proved, for thus the the head would catch some of the eastern as well as the northe.n electricities. Some robost persons and especially ]aborors who do not use their brains much, can sleep in all directions with comfort, and yet many of these will after awhile get their nervous systems unduly excitable by sleeping wrongly.

FRICTIONAL ELECTRICITY and also heat combined may be aroused by making passes of the hand over the skin, by the use of the flesh brushes, coarse towels, spatting with the hands, muscle beating with an elastic flat or whalebone, pinching and many other methods. The hand is quite superior to any artificial instruments as it conveys a magnetic life power to the patient besides arousing the frictional electricity. Mere frictional electricity is a coarse grade of power and flows along so near the surface of the body where the nerves are sensitive that it is rather exciting to very nervous persons and yet is of great value in arousing the skin to action and relieving internal organs which may be inflamed or overburdened. The hand of a developed magnetist is much more powerful than that of ordinary persons, but all persons have some power.

THERMAL AND ELECTRICAL SUBSTANCES. The following substances have a predominance of heat: the red, orange, yellow and yellow-green colors of sunlight, the alkalies, the sweets, most of the metals, and most substances which are red, orange and yellow, or which in the spectroscope have a predominance of these colors. Hydrogen is the greatest heat element, being of a brilliant red in the spectroscope, and carbon abounds in heat and has the yellow-orange principle prominent, although it is also somewhat electrical. The cold or electrical principle predominates in the blue-green, blue, indigo, and violet colors of sunlight, in electricity of the battery, in acids, in substances which are innately blue or violet or blue-green, etc. Oxygen is the most electrical of atoms and the spectroscope gives blue and indigo as its leading power. It should be remembered that by chemical processes a cold element like electricity may develop the greatest heat known by exciting a warm element into action; that the blue, cold element of oxygen may intensify the heat of the blood by arousing the hydrogen and other red principles into activity through chemical affinity; that a dash of ice water will set a person into a glow of warmth if there are warm elements within to answer to it. A large quantity of cold elements, however, will be apt to draw off the heat and give coldness, as cold water applied for some time would do. General Pleasanton, of Philadelphia, had one-eighth of the panes of glass in his grapery blue and the rest transparent, and caused the heat inside to rise to 100° Fahr. when it was only 36° on the outside. If he had put half or more of the panes blue, it would have been colder than it would with all the panes transparent, although blue or dark colored glass itself is always warmer while receiving the sun's rays than transparent glass.

